# Impact of Dust from Ore Processing Facilities on Rain Water Collection Tanks in a Tropical Environment—The Obvious Source “Ain’t Necessarily So”

**DOI:** 10.3390/ijerph13020243

**Published:** 2016-02-20

**Authors:** Brian Gulson, Michael Korsch, Anthony Bradshaw

**Affiliations:** 1 Department of Environmental Sciences, Macquarie University, Sydney, NSW 2109, Australia; 2 Commonwealth Scientific and Industrial Research Organisation (CSIRO), Energy Flagship, Sydney, NSW 1670, Australia; mike.korsch@gmail.com; 3 Environmental Services & Regulation, Queensland Department of Environment & Heritage Protection, Brisbane, QLD 4000, Australia; tony.bradshaw@ehp.qld.gov.au

**Keywords:** waters, dust, processing, shipping, lead, isotopes

## Abstract

Concerns have been expressed that dust from the minerals processing facilities at Karumba Queensland Australia have resulted in elevated lead (Pb) concentrations in rain water tanks. The ores derived from the Century mine some 304 km from the port. High precision Pb isotopic measurements on environmental samples have been undertaken to evaluate the source of Pb in rainwaters and acid digests from roof wipes and gutter wipes. There does not appear to be any relationship between sample location and the processing facility but samples from the area subject to the prevailing winds show the highest contribution of Century Pb. All gutter wipes (82 to 1270 µg Pb/wipe) have contributions of Century ore ranging from 87% to 96%. The contribution of Century ore to five roof wipes (22 to 88 µg Pb/wipe) ranges from 89% to 97% and in the other two samples there is a mix of Century and Broken Hill Pb. Three of the seven rainwater have contributions of Century ore Pb ranging from 33% to 75%. Two of the other four rainwater samples have the highest water Pb concentrations of 88 and 100 µg/L and their isotopic data show Broken Hill Pb contributions ranging from 77% to 80%. The source of the Broken Hill Pb is probably from the galvanized roofing material and/or brass fittings in the rainwater tanks. The discrimination between various sources is only detectable using high precision ^204^Pb-based isotopic ratios and not the now common inductively coupled plasma mass spectrometry (ICP-MS ) data presentations of the higher abundance isotopes ^208^Pb, ^207^Pb and ^206^Pb. Isotopic results for the waters demonstrate that apportioning blame where there is an obvious point source may not always be the correct conclusion. Nevertheless the isotopic data for the gutter wipes indicates that there was widespread contamination from the processing facilities throughout the town.

## 1. Introduction

Ore processing and handling facilities are commonly located in harbours where potential exposure of urban neighbourhoods can occur. Some examples where contamination arising from bulk mineral rail transport and port unloading and loading has taken place include Callao Peru [1], Alaska [2], Russia [3], Esperance in Western Australia [4,5] and Townsville Queensland Australia [6].

**Figure 1:**
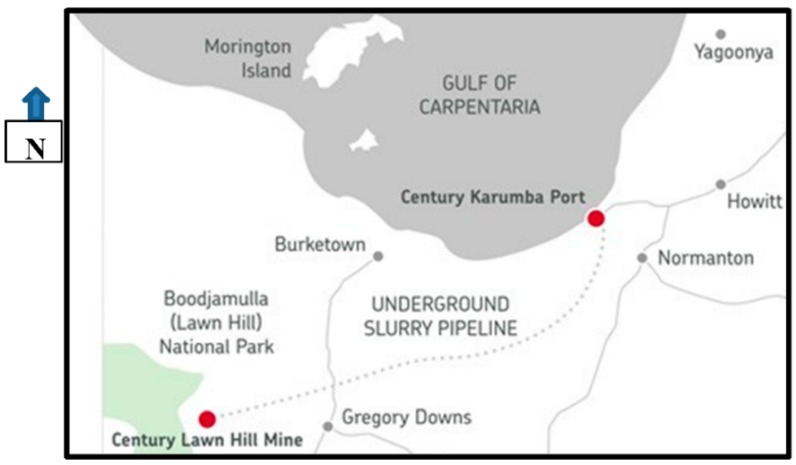
Map showing location of OZ Mineral’s Century Mine, the slurry pipeline and the Karumba Port Operations. The pipeline runs for 304 km.

OZ Minerals Century Limited (OZ Minerals) operates a mineral concentrate processing and marine loading facility in Karumba Queensland for export of zinc (Zn) and lead (Pb) concentrates from the Century Mine (see Figure 1, and Supplementary Materials Figure S1). During its 16 years of operation with the first shipment in 2009, Century has produced and processed Zn and Pb concentrates at Lawn Hill. The product has been transferred in slurry form via a 304 km underground pipeline to Century’s port facility at Karumba for shipping to smelters in Australia, Europe and Asia. On occasions, Pb concentrate has also been trucked from the mine to the Karumba Port site. The final ore was extracted from the Century open pit in August 2015. Century will end all production and shipping by the end of 2015 [7].

At Karumba, the concentrate slurries are dewatered, the Zn concentrate and sometimes Pb concentrate dried in an oil-fired rotary dryer and then stored in a large building. Concentrates must be kept below a specific transportable moisture limit to allow safe shipping. The concentrates are exported by transferring 5000 tonne loads via an enclosed conveyor to a shallow draft self-loading and discharging vessel (Supplementary Materials Figure S2). The vessel docks beside the OZ Mineral’s port-site facility on the Norman River and transfers its loads to much larger export vessels in deeper water about 45 kilometres offshore.

Karumba, which is located in the Gulf of Carpentaria region of Queensland, experiences summer monsoonal wet seasons and typically arid conditions for the remainder of the year. Rain water tanks are a historic and now secondary source of potable water in the town. Given the arid climate, the residents value having a secondary supply of water and some have expressed a preference for the rainwater based on taste and aesthetics. Concerns have been raised that emissions of concentrate dust from the loading facility have given rise to varying metal concentrations, especially Pb, in rain water tanks. Lead values of up to 100 µg/L have been measured in tanks and these values compare with the WHO and Australian National Health and Medical Research Council guidelines of 10 µg/L.

The aim of this study was to determine the source(s) of Pb in potable water samples obtained from rain water tanks, from gutter wipes and roof wipes, and ambient dust in Karumba using high precision Pb isotopes.

## 2. Experimental Section

### 2.1. Sampling

**Figure 2:**
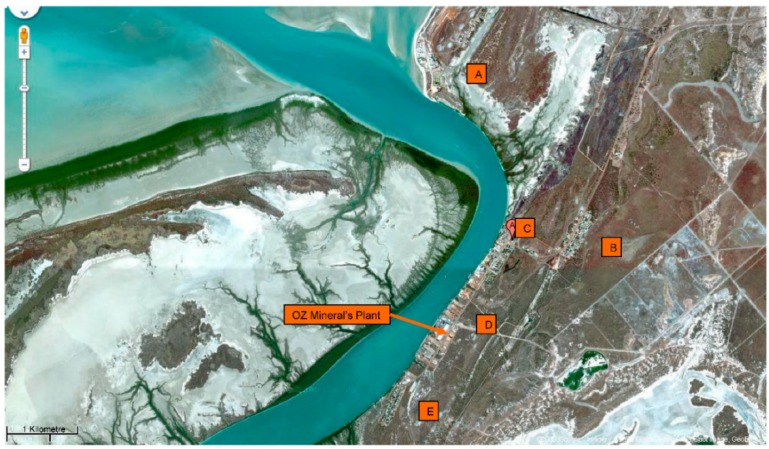
Map of Karumba Area showing OZ Mineral’s Mineral Processing and Export Facility and the localities of Karumba Point (**A**); Henry Street Area (**B**); Town Centre (**C**); Yappar Street Area (**D**) and Highbanks (**E**) (Source: Google Maps 2015).

These samples were taken over 21 to 23 October 2008 (Figure 2). The sampling period at the end of the dry season was chosen as it was considered that, absent of a large scale emission event, the greatest particulate loadings on roof and gutter surfaces would occur over this period. Rainfall figures are given in Supplementary Materials Table S1. Particulate Pb surface loadings were considered an indicator of risks to tank water quality. It was understood that resultant Pb concentrations in the collection tanks would be influenced by other factors such as tank material, the volume of rainfall that collected in the tank and hence degree of dilution, presence of and effectiveness of any first flush diversion device fitted to the tank inlet, and the degree to which particulate matter, settled or dissolved, in the tank. As mentioned above, the key aim of the investigation was to ascertain whether the ore processing facility was a material contributor to the elevated Pb concentrations previously measured in the tanks, which was a concern to the local community.

The tanks were of variable volume, constructed usually of PVC and galvanised steel with some made of fibreglass (Supplementary Materials Figures S3–S6). Fittings were brass and plastic. Other parameters which could influence the Pb concentrations such as volume of water remaining when sampled, residence time of water and frequency of use were variable and dependent on individual use so were not measured. Downpipes directed rainwater from gutters into tanks (e.g., Supplementary Materials Figures S3–S6).

The roof and gutter sampling utilized dust wipes and plastic specimen jars. The jars were obtained from the Queensland Health Scientific Services Laboratory, at Nathan in Brisbane. The wipes used were proprietary 15 cm by 15 cm “Ghost™ wipes” produced by Environmental Express. These surface wipes meet ASTM E 1792 “Standard specification for wipe sampling materials for lead in dust”. Also used were two large 61 cm by 40.5 cm (external sides) metal framing squares that were marked with 2 mm gradations (Supplementary Materials Figure S3B). Surgical gloves were worn during sampling to minimise the chance of contaminating the wipe with material of the sampler’s hand.

Roof Samples—Roof areas were a combination of galvanized iron (Supplementary Materials Figure S3B), zinc aluminium alloy and Colorbond steel (Supplementary Materials Figure S4B). The framing squares were first cleaned by wiping any externally visible dust off with non-aloe “nappy wipes”. To obtain a sample, the framing squares were placed flat down on the surface to be sampled and across each other, to create a 30 cm by 30 cm template using the internal edges of the squares. For initial samples, one sampling team misread markings on the squares and so for a small number of samples, the area sampled was 21 cm by 30 cm. These were samples KPR2, KSR1, KHR1, KHR2 and KHR3. This reduced area was taken into account in calculating loads. The wipe was taken out of its sealed packet and used to wipe up any dust particles onto the pre-moistened wipe. The wipe containing the dust was transferred into a small screw top plastic specimen jar.

Gutter Samples—Gutter samples were taken using the same types of wipes as used for roof sampling, but a 50 cm length of gutter was wiped instead to remove readily wipeable sediment that had collected in the gutter. The breadth of the gutter was typically 120–160 mm, depending upon the gutter’s profile. No gutter sample was taken from taken from 4 Ward Street Karumba (Supplementary Materials Figure S3) as it was not possible to wipe the inside of the gutter due to the roof sheet overhang not allowing sufficient space to obtain a sample. A roof sample only was taken from this site.

The other exception to the general sampling methodology was for sample KHG2, where leaf debris was collected from a 50 mm length of gutter. This was necessary as there was a build-up of leaves in the gutter. It was assumed that the leaf litter would host dust fall e.g., that which fell onto the nearby trees and then into the gutter.

Blank Samples—A number of trip blank and field blank samples were taken, including a field blank for each day’s sampling. These were analysed for arsenic, cadmium, lead and zinc.

Rinsate Samples—A rinsate sample using laboratory prepared distilled water was taken off the squares on each day surface sampling was undertaken. Distilled water was supplied by Queensland Health Scientific Services. The rinsate was analysed for arsenic, cadmium, lead and zinc.

Ambient dust samples—Two ambient dust samples were taken at distances of approximately about 12 and 40 km from Karumba in the easterly wind direction and were sampled to provide background isotopic data. Wind roses for the Karumba airport recording station show that easterly winds represent approximately 19% of the 9 a.m. and 9% of the 3 p.m. wind recordings respectively (Supplementary Figures S7 and S8). Samples were collected at each site using a new fine-haired paint brush to lightly brush, from the surface, loose dust particles onto a folded filter paper. Six sub-samples of dust were collected and transferred into a screw top plastic specimen jar. A fresh brush was used for each sample site.

Water tank samples—were taken in acid-washed plastic bottles, with contents acidified in the field to pH 1–2 with 70% m/V nitric acid. Samples were taken from tank outlets after allowing the water to run clear. As a control on sampling procedures, two container trip blank and one field blank samples were analysed for Pb concentration (Table S2). These samples were obtained by adding reverse osmosis water and the nitric acid preservative to the water sample containers.

### 2.2. Methods

Roof and gutter wipe samples were sub-samples of acid digests from previously analysed samples. The digests were provided by Queensland Health Scientific Services (QHSS), which carried out the digestion according to Australian Standard *AS 4479.2-1997:* “Analysis of soils—Extraction of heavy metals and metalloids from soil by aqua regia—Hotplate digestion method”. Only those samples where water quality results indicated a breach of Pb drinking water standards were analysed for Pb isotopic composition.

The net Pb loadings of the roof surfaces were calculated as µg Pb/m^2^ and net Pb loadings of the gutter surfaces were calculated as µg Pb/m of gutter. There were a small number of instances in which heavy soiling or very hot surfaces (these rapidly dried out the pre-moistened wipe upon contact) necessitated use of more than one wipe to obtain sample. In these cases, net loading was calculated via total mass of metal—(mass of metal in a field blank swipe by number of wipes used to obtain sample). These samples are: QHFSS sample numbers 08NA10511 (sample PSTB1008G3) and 08NA10512 (sample PSTB1008G4)—used four wipes in each; and QHFSS sample number 08NA10516 (sample PSTB1008RF4)—used two wipes.

Calculated Pb loadings for roof samples are given in Table 1, together with total Pb concentrations measured in rainwater tanks on site.

#### 2.2.1. Pb Isotope Method

The Pb isotope method makes use of the variations, arising from radioactive decay throughout geological time, in abundances of three of four Pb isotopes, the relative concentrations of Pb, Th and U, and the time when the ore was formed. Lead has four naturally-occurring isotopes three of which are the stable end products of radioactive decay of uranium and thorium. For example, ^206^Pb is derived by radioactive decay from ^238^U with a half-life of about 4500 million years, the decay of ^207^Pb from ^235^U is more rapid with a half-life of about 700 million years, and the decay of ^208^Pb from ^232^Th is even longer at about 14,000 million years. The other low abundance isotope, ^204^Pb (approximately 1%), has no known radioactive parent and is thought to represent Pb present at the time of formation of the earth, about 4550 million years ago (so-called primordial Pb); it is used as a reference isotope. Hence Pb mineral accumulations (mines, deposits) of different geological age have different isotopic abundances. The simplest explanation for the different abundances is that when the deposit formed, the Pb was separated from its parent Th and U isotopes and so no further radioactive decay or changes in the isotope abundances occurred. In this way, ore formed say 1700 million years ago, such as the Century or Mt Isa deposits, had more primordial Pb and relatively less decay products compared with ores formed only 400 million years ago; this also includes Pb formed in the intervening 1300 million years.

**Figure 3:**
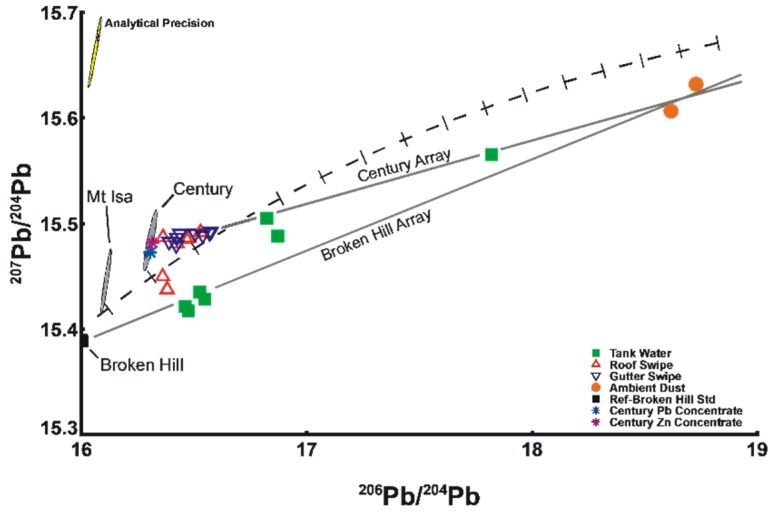
Isotopic ratio plot of the uranium derived Pb isotopes ^207^Pb/^204^Pb *versus* ^206^Pb/^204^Pb showing the resolution between the Century and Broken Hill sources in water samples. The conservative precision of the isotopic measurements is shown by the 95% confidence ellipse in the upper left hand corner. The dashed line is the Growth Curve or Pb Evolution Curve for massive sulfides. The ellipses for Century and Mt Isa are drawn in grey and the value for Broken Hill denoted by the filled square. Lines have been drawn between the Century field and the background ambient dust sample data points, termed the Century Array. A similar line can be drawn through the Broken Hill value and the background data points (the Broken Hill Array).

The data are presented as ratios of abundance of one to the other, such as ^208^Pb/^204^Pb (also called “thorogenic” isotopic ratio), ^207^Pb/^204^Pb, and ^206^Pb/^204^Pb (“uranogenic” isotopic ratios), or any combinations of these. In the earlier days of use of Pb isotopes, because of the difficulty in measurement of the low abundance ^204^Pb isotope, the data were reported as ^207^Pb/^206^Pb (or the inverse ratio) and/or ^208^Pb/^206^Pb ratios. This is the common presentation these days as much Pb isotopic data are obtained by ICP-MS which has problems with measuring ^204^Pb. As an example of the differences in isotopics from different ore deposits, the geologically-ancient so called massive sulfide lead-zinc-silver Broken Hill and Mt Isa deposits formed about 1700 million years ago and have a ^206^Pb/^204^Pb ratio of 16.0 or 16.1 respectively, whereas geologically younger deposits of similar mineralogical composition in eastern Australia that formed 400 to 500 million years ago have a ^206^Pb/^204^Pb ratio of about 18.1. In Figure 3 the value for the Broken Hill deposits is shown by the solid square. It has been found that the Pb isotopic data for the majority of massive sulfide deposits lie on a so-called Pb evolution or Growth Curve (the dashed line in Figure 3) which represents the changes in isotopic ratios over geological time (e.g., [8]).

#### 2.2.2. Analytical Methods

Details of these methods are given in the Supplementary Materials.

## 3. Results and Discussion

Samples analysed for Pb isotopes are listed in Table 2 and Table 3 along with metal concentrations, and metal loadings for roof and gutter samples compared with rain water Pb values are given in Table 1. The Pb isotopic results are listed in Table 4 and plotted on isotope ratio diagrams in Figure 3 and Supplementary Materials Figure S9. Isotopic data may be presented in a variety of ways but the ^207^Pb/^204^Pb *versus* ^206^Pb/^204^Pb graph (Figure 3) is the most illustrative for the current study and allows discrimination of sources whereby other types of plots may not, as shown in Supplementary Materials Figure S9.

### 3.1. Blank Samples

The concentrations of Pb for the blank containers are 3.4 and 1.8 ng and the field blank sample are 19 ng Pb. The low values have an insignificant effect on the measured ratios.

### 3.2. Ore Concentrates

The isotopic results for the samples of Zn and Pb concentrate are the same to within experimental error and are also the same as earlier unpublished CSIRO analyses of sulfides from the Century deposit (Figure 3; Table 4). The ^207^Pb/^204^Pb ratios lie slightly above the Growth Curve and are significantly different from those for Mt Isa and would possibly allow for discrimination of these two sources, be they Pb or Zn (Figure 3).

### 3.3. Ambient Dust

The Pb contents of Karumba soils have been monitored in the range of <5 to 200 mg/kg [7]. The isotopic ratios for the two ambient dust samples, considered to represent background values, are similar and the small difference between results is attributed to time-dependent fractionation of the isotopes during the mass spectrometer measurement (the lighter isotope ^204^Pb is “distilled” at a faster rate than the heavier ones).

A combined isotopic value of these two samples is taken to be the background value and used to estimate proportions of Century Pb and background Pb or, alternatively, Broken Hill and background Pb in the rainwater and wipe samples, as described in the following section.

### 3.4. Roof and Gutter Wipes

Apart from two samples of roof wipes, the other isotopic data lie in a small cluster along a well-defined mixing line between the Century ore and the background ambient dust samples (termed the “Century Array”). The data for some rainwater samples lie on another array which is defined by the isotopic data for end-members Broken Hill and the background samples (termed the “Broken Hill Array”). It is possible to calculate the contributions of Pb in the wipes and rainwater samples from these two pairs of different sources using well-established methods from isotope geochemistry assuming two component mixing [9]. Any point on a graph of isotopic ratios can represent a single homogeneous source or it can be a mix of two or more isotopically different sources. When Pb from two sources is mixed, the resultant isotope value will lie on a straight line between the end members. The distance along that line is proportional to the ratio of the amounts of Pb from each of the sources. Thus a 50:50 mix will give a point half way along that line. More complex mixing relationships are possible [10] but it is not necessary to resort to such explanations in the present case. The proportions of Pb from Century and Broken Hill type ore are also listed in Table 4.

Century Pb was found in all gutter dust samples from the different localities with amounts ranging from 87% to 96%. Apart from the samples whose data are highlighted in italics, the contributions from Century Pb for the roof wipe samples range from 89% to 97%. The two aberrant roof wipe samples have data lying slightly below the Century Array and towards the Broken Hill Array and come from residences with galvanized zincalume roofs which show evidence of oxidation. It is highly likely that the zinc used in the galvanizing was sourced from the Broken Hill mines and/or Woodlawn mine [11] and these Pb’s are a mixture with those from Century.

There does not appear to be any correlation between the amounts of Pb in the wipes and the isotopic composition/percentage contribution from Century or Broken Hill type (data not presented).

### 3.5. Rainwater Tank Samples

Lead concentrations in the rainwater tank samples range from 13 to 100 µg/L. Only three of the samples appear to have significant contributions of Century Pb ranging from 33% to 75% (equivalent to 9–32 µg/L in the respective water samples, the latter being three times greater than the WHO guideline). The data for the sample with the lowest contribution (W966) and possibly W970 could lie on either the Century Array or the Broken Hill Array. There is no correlation between the amounts of Pb in the rainwater and the isotopic composition/percentage contribution from Century or Broken Hill Pb (data not presented).

Four of the rainwater samples lie on the Broken Hill Array, as could the data for sample W966. The contributions of Broken Hill Pb range from 77% to 80% and that for W966 is similar to the value for the Century contribution (Table 4). The tank contents were accessed via brass tap fittings. It is probable, as in other areas, that Broken Hill/Mt Isa Pb was used in the manufacture of the plumbing materials [12,13]. In addition the high temperatures of this area may have enhanced the solubility of Pb from the brass fittings [14].

The data for the rainwater sample W970 from Karumba State School could also lie on both arrays but the data for both roof wipes (W979) and gutter wipes (W988) from the school lie on the Century Array. This indicates there is an additional source of lead contaminating the tank rainwater rather than that in the dust. Roof, tank or plumbing materials as described above are suggested.

### 3.6. Lead Isotopic Results and Location of Environmental Samples

The locations of the samples are also listed in Table 1. It is not possible to state anything definite about distance from the processing facility because of the small number of samples and because four out of seven rainwater samples appear to have a significant contribution of Broken Hill Pb. However, five wipe samples with the lowest ^206^Pb/^204^Pb ratios and hence highest proportion of Century Pb are from area E, the Highbanks area south-west of the processing facility and the dominant prevailing wind direction in the afternoon (Figure S8).

### 3.7. Comparison with Other Similar Studies

Highly elevated blood Pb levels (mean 25.6 µg/dL, *n* = 314) were found in children from schools located in Callao Peru, the port in which Pb and zinc mineral concentrates were stored prior to shipment. In a comprehensive Pb isotopic investigation of blood, mineral concentrates, gasoline and air particulates, Naeher *et al.* [1] found that the primary source of Pb in the children was from the mineral concentrates. They also showed that dust from stockpiles at the port was transported at least 15 km based on air particulates collected in the capital Lima.

In a novel investigation of the transport, loading and unloading of bulk Pb-Zn and nickel concentrate through the port of Townsville northern Queensland Australia, Taylor and students [6] measured arsenic, cadmium, Pb and nickel in hand wipes simulating pre-play and post-play conditions. They found that mean Pb in post-play handwipes (965 µg/m^2^/day) was more than 10 times the mean pre-play loadings (95 µg/m^2^/day) and that repeat sampling over a 5-day period showed that hands and surfaces were recontaminated daily. Lead isotopic ratios were measured by ICP-MS on post-play handwipes for day 1 (*n* = 4) and day 5 (*n* = 4). The ratios in three of the four day five wipes were similar to the values for the Mt Isa ores that are exported through the port. The results for the other five wipes were totally different but no interpretation of these results was offered. They may reflect the contribution from the nickel ores, nickel being elevated in the wipes, although the feedstock to the Greenvale smelter has a complex history with lateritic nickel product not only coming from the local mine but also from south East Asia [15].

Lead carbonate concentrate handling resulted in contamination of the coastal township of Esperance in Western Australia. Fortunately the community was alerted to the problem because of birds dying of Pb poisoning. A multi-agency group, the Esperance Cleanup and Recovery task force was established to test for Pb and nickel contamination and monitor the success of cleanup and involved extensive environmental testing including roof wipes and ceiling voids (e.g., Esperance Cleanup and Recovery Project Update March 2011). In addition, an ongoing blood testing program was instigated. High precision Pb isotopic ratios were used to evaluate the source of Pb in environmental and blood samples for use in legal proceedings, and for use in remediation and monitoring. Isotopic measurements were undertaken of bird livers, plants, drinking water, soil, harbour sediments, air, bulk ceiling dust, gutter sludge, surface swabs and blood. The unique Pb isotopic signature of the contaminating Pb carbonate enabled diagnostic apportionment of Pb in samples. Blood results demonstrated all children less than five years of age had significant proportions of the ore Pb concentrate in their blood, in spite of the PbB levels being quite low, with the majority being <5 µg/dL. This provided conclusive evidence that the source of Pb contamination of the Esperance community was from the carbonate concentrate and consequently shipping of the concentrate through the Port was suspended in March 2007 after less than two years of operation. Apart from some soil and water samples, the proportion of contaminating Pb was >95% in the environmental samples. Lead isotopes were critical in resolving legal proceedings, were used in the remediation of premises, and in monitoring of workers involved in the decontamination of the storage facility [4,5]. Roof and gutter samplings were undertaken in the early stages of the cleanup in Esperance but because of the absence of a guideline Pb value for such wipes, variable results, and no strong link between gutter sludge, rainwater tank and roof surface results, such sampling was discontinued (Matt Devenish, personal communication, 2015). Nevertheless gutter sludge data for 45 houses showed that 73% were above soil guidelines of 300 mg/kg; 83 houses had roof surface wipes ranging from 0 to 7 µg/cm^2^ with 69% showing values less than 0.4 µg/cm^2^ and 31% with values greater than 0.4 µg/cm^2^ ,equivalent to 4000 µg/m^2^ (Esperance Cleanup and Recovery Project, Minutes of Steering Committee Meeting 11 March 2010). None of the roofs in Karumba had such high values but comparisons of deposition between communities depend on the time interval of deposition and weather conditions.

Roof and gutter dusts were measured in buildings around the Port Kembla area, south of Sydney, and a location of steel works, a former copper smelter and a former coal-fired power station. Lead concentrations ranged from 275 to 3275 mg/kg and high precision Pb isotopic measurements indicated that an air-gasoline component contributed from 60% to 92% of the Pb in the dusts [10].

The contribution of Broken Hill Pb to several rain water tanks rather than Century Pb is similar to the case in the Broken Hill mining community where comprehensive Pb isotopic investigations showed that although the mine Pb was the dominant contributor to blood leads in children and some adults, there were others in which gasoline and paint Pb were also sources of elevated blood Pb [16,17].

### 3.8. Potential Health Impacts

Although the main aim of this study was to evaluate the source of Pb in rainwater from residential tanks and dust on roofs, the ultimate aim was to determine the potential impact of the Pb in water on health especially in children. Although no blood samples were collected from children, an estimate of their potential exposure from the water can be obtained by employing the US EPA Integrated Exposure Uptake Biokinetic Model (IEUBK) for children [18].

Modelling using the lowest and highest concentrations of Pb in the water of 13 and 100 µg/L respectively and default values for other exposure media gives blood Pb values for a 2–3 year old of 4.0 and 10.1 µg/dL. The maximum value of 10.1 µg/dL exceeds the newly recommended guideline of 5 µg/dL, a level above which investigation of the source of Pb should be undertaken [19]. However the rain water samples with the highest amount of Pb (88 and 100 µ/L) have large contributions of Broken Hill type Pb rather than Century Pb. In this case study the source is known and in residences where rain water is the primary drinking supply, further investigation is warranted. A similar situation was experienced in Esperance, described above, where the multi-agency Esperance Cleanup and Recovery Project undertook comprehensive sampling and recommended that the tanks and especially residue in the tank bottoms were flushed on a regular basis (Esperance Cleanup and Recovery Project Update March 2011). In addition, the council supplied the community with bench top water filter appliances which had been shown to efficiently remove Pb from varied Australian water samples [20].

## 4. Conclusions

In response to the roof and gutter wipe results along with the Pb isotopic measurements the company reviewed all potential sources and implemented additional dust mitigation measures to minimise emissions. They also upgraded their dust monitoring program with additional fallout bottles and improved QA on analysis and monitoring. The company were receptive to implementing best practice and engaging with and looking after the community.

The majority of the dust obtained from the roof and gutter wipes has an overwhelming contribution from Century Pb. In contrast, four out of seven of the rainwater samples have a significant contribution probably from Broken Hill Pb. Two of the remaining three rainwater samples could also have Broken Hill Pb or this could also be from Century or Mt Isa Pb.

Even though most gutter and roof samples show the presence of Century Pb and would normally be considered a potential risk to water quality, the fact that only three of the water samples analysed in the present study indicate the presence of Century Pb might suggest that simple explanations of solubilisation of the concentrate in the rainwater are not valid. Nevertheless, the results of modelling show that water samples with Pb values of 100 µg/L could potentially give rise to blood Pb’s in young children that exceed the new guidelines.

Alternatively, the unexpected isotopic differences between the rainwater samples and roof and gutter samples may suggest that the tank or roof type could play an important role. This could be in solder in the tank joins, if it is of galvanised iron construction, and/or the tap if brass or other metal. Such factors can be important in determining the source of the Pb in the drinking water as we have shown in other studies [12,13]. If the tank is of polypropylene or other plastic, these may also contain traces of Pb but the tap could also contribute Pb if it is brass. As temperature and pH play an important role in the metal content of water, these could be additional contributing factors [14]. Rainwater commonly has a lower pH than neutral. Temperature and pH were not measured. Roof and gutter construction and condition could also play an important role and where Broken Hill Pb is present, the Pb may be released to the rainwater.

Another possible explanation apart from the contributions from tank, tap and roof/gutter materials could be the rainfall records. These show that there was no rain in the Karumba area from 14 March 2008 to 7 October 2008, when 5 mm fell (Supplementary Materials Table S3). No rain was recorded until mid-December 2008. Queensland EPA sampled roof, rain water and gutters in late October, so it may be that the 5 mm of rainfall was insufficient to mobilise the total roof and gutter dust burden (including any Century Pb) into the tank in quantities that would overwhelm the relative Pb contributions from materials in the roof and tank.

This study shows that high precision Pb isotopic analyses by TIMS are a useful environmental management tool in determining relative contributions in this particular setting, e.g., if one needed to discriminate between ambient, Century and other Pb sources to ascertain Century Pb contributions in environmental or human or animal samples. Furthermore, the evidence that there is a contribution of Pb from Broken Hill or Mt Isa mines demonstrates that just because there is an obvious point source of lead contamination, that is the ore processing facility, does not necessarily translate into it being the source of Pb in all other samples.

## Figures and Tables

**Table 1:** Comparison of net Pb loadings on roof surfaces with total lead concentrations measured in rainwater tanks at sampling locations.

Sample Location (Figure 2)	Roof Surfaces Net Load Pb µg/m^2^	Gutter Surfaces Net Load Pb µg/m	Tank Water 1+	Tank Water 2+
12 Riverview Road (E)	834	652	23	-
77 Karumba Development Rd (B)	657	1270	<5	-
83 Karumba Development Rd (B)	557	749	9	88
85 Karumba Development Rd (B)	8.9	2530	36	<5
8 Palmer St Karumba Point (A)	106	348	Tank empty	-
27 Riverview Drive, Highbanks (E)	50.8	4.8	100	-
19 Riverview Drive, Highbanks (E)	335	198	8	-
Barra Farm, Highbanks (E)	1380	450	<5	-
Karumba School (C)	367	162	13	-
4 Ward Street Karumba Point (A)	734	NM	Tank empty	-

+ The residence was serviced by two tanks.

**Table 2:** Rain Water Tank Water Samples.

EPA Sample Number	Locality on Figure 2	Contaminant Location	Results			
			Cd µg/L	Pb µg/L	As µg/L	Zn mg/L
CIBJB1008-W1	Henry St Area	12 Riverview Drive	**3**	**23**	**<5**	**2.0**
CIBJB1008-W4	Henry St Area	Tank B-83 Karumba Development Rd	**<1**	**88**	**<5**	**10.8**
CIBJB1008-W5	Henry St Area	85 Karumba Development Rd	**<1**	**36**	**<5**	**0.16**
KHW1	Highbanks Area	27 Riverview Drive, Highbanks	**<1**	**100**	**<5**	**0.81**
KDW1	Yappar Street	Council Depot, Yappar Street	**6**	**42**	**<5**	**1.3**
KCW1	Yappar Street	Gulf Caravan Park, Yappar Street	**<1**	**22**	**<5**	**1.1**
KSW1	Town Centre	Karumba State School	**<1**	**13**	**<5**	**0.76**

**Table 3:** Acid digests from roof and gutter surface wipe samples.

Sample Location	Locality on Figure 2	µg/Wipe			
		As	Cd	Pb	Zn
**Roof Wipes**					
12 Riverview Drive	Highbanks Area E	<20	<2	76	7790
77 Karumba Development Rd	Henry St Area B	3.7	0.57	60	496
83 Karumba Development Rd	Henry St Area B	4.7	0.33	51	377
19 Riverview Drive	Highbanks Area E	<2	<0.2	22	443
Barra Farm	Highbanks Area E	<4	0.4	88	997
Karumba School	Town Centre C	3.3	0.22	24	167
4 Ward St	Karumba Point A	3.9	0.42	67	339
**Gutter wipes**					
12 Riverview Drive	Highbanks Area E	<80	18	327	36829
77 Karumba Development Rd	Henry St Area B	<40	5.3	635	13930
83 Karumba Development Rd	Henry St Area B	<100	<10	378	9012
85 Karumba Development Rd	Henry St Area B	<200	<20	1270	11610
19 Riverview Drive	Highbanks Area E	6.4	1.1	100	2600
Barra Farm	Highbanks Area E	<8	2.1	226	3780
Karumba School	Town Centre C	4.8	0.75	82	738
8 Palmer St	Karumba Point A	12.4	1.9	175	2390

**Table 4:** Lead isotopic compositions and Pb contents for samples.

Tank Water			^206^Pb/^204^Pb	^207^Pb/^204^Pb	^208^Pb/^204^Pb	Pb (µg/L)	% Century	% Broken Hill	Locality
*1*	*W964*	*CIBJB1008-W1*	*16.546*	*15.428*	*36.175*	*23*		*77*	B
*2*	*W965*	*CIBJB1008-W4*	*16.461*	*15.422*	*36.139*	*88*		*80*	B
3	W966	CIBJB1008-W5	17.820	15.565	37.716	36	33		B
*4*	*W967*	*KHW1*	*16.475*	*15.417*	*36.133*	*100*		*79*	*E*
5	W968	KDW1	16.822	15.505	36.547	42	75		D
*6*	*W969*	*KCW1*	*16.524*	*15.435*	*36.215*	*22*		*78*	*D*
7	W970	KSW1	16.871	15.488	36.640	13	72		C
**Roof wipes (µg Pb/wipe)**									
*1*	*W974*	*PSTB1008-Rf1*	*16.360*	*15.450*	*36.038*	*76*	*95*	*86*	*E*
2	W975	PSTB1008-Rf2	16.476	15.486	36.210	60	91		B
3	W976	PSTB1008-Rf3	16.528	15.492	36.267	51	89		B
4	W977	KHR2	16.464	15.486	36.176	22	92		E
5	W978	KHR3	16.361	15.487	36.078	88	97		E
6	W979	KSR1	16.424	15.481	36.133	24	94		C
*7*	*W980*	*KPR1*	*16.380*	*15.438*	*36.067*	*67*	*94*	*85*	*A*
**Gutter wipes (µg Pb/wipe)**									
1	W981	PSTB1008-G1	16.421	15.480	36.142	327	94		E
2	W982	PSTB1008-G2	16.503	15.490	36.228	635	90		B
3	W983	PSTB1008-G3	16.568	15.491	36.303	378	87		B
4	W984	PSTB1008-G4	16.574	15.492	36.296	1270	87		B
5	W985	KPG2	16.538	15.489	36.269	175	89		A
6	W986	KHG2	16.423	15.487	36.145	100	94		E
7	W987	KHG3	16.388	15.483	36.094	226	96		E
8	W988	KSG1	16.436	15.490	36.167	82	94		C
**Ambient dust**									
1	W991	PSTB1208-D1	18.617	15.607	38.584	12 km from Karumba			
2	W992	PSTB1208-D2	18.728	15.632	38.632	40 km from Karumba			
**Ore samples**									
		Broken Hill	16.002	15.389	35.657				
	W989	Century Pb concentrate	16.306	15.473	35.987				
	W990	Century Zn concentrate	16.314	15.482	36.012				
		Mean Century ore (*n* = 10)	16.306	15.483	36.013				

The water data in italics plot on the Broken Hill Array. The data for the two roof wipes lie between the Century and Broken Hill arrays.

## Supplementary Materials

The following are available online at www.mdpi.com/1660-4601/13/2/243/s1, Table S1: Rainfall at KarumbaAirport, 2008. Table S2: Container Blanks. Table S3: Estimated composition of the concentrate products (Source: OZ Minerals). Figure S1: OZ Mineral’s Port Site Processing, Storage and loading Facility located beside Norman River at Yappar Street, Karumba. Figure S2. MV Wunma steaming out of Norman River to offshore anchorage where concentrates are transferred to export vessels. Figure S3: (A) 4 Ward Street Karumba point tank and house; (B) Roof at 4 Ward Street; (C) Tank at 4 ward Street, Fibreglass discharging through galvanised water pipe and brass tap fitting; (D) Checking pH of sample following addition of HNO3 preservative. Figure S4**:** 12 Riverview Drive Karumba. (A) PVC tank; (B) Colorbond roof; (C) PVC downpipes and tank. Figure S5**:** Rain water tank at Karumba School. Figure S6**:** Rainwater tank council depot. Figure S7**:** Summary of 9 a.m. Wind Data for Karumba Airport. Figure S8**:** Summary of 3 p.m. Wind Data for Karumba Airport. Figure S9**:** Isotopic ratio plot of the major isotopes ^207^Pb/^206^Pb *versus* ^208^Pb/^206^Pb showing the lack of resolution in data using this plot.
