# Supplementary material for: Impact of Dust from Ore Processing Facilities on Rain Water Collection Tanks in a Tropical Environment—The Obvious Source “Ain’t Necessarily So”
Source: Int J Environ Res Public Health. 2016 Feb 20;13(2):243. doi: 10.3390/ijerph13020243 (PMC4772263; doi:10.3390/ijerph13020243)
Supplement: Supplementary file 1 [file ijerph-13-00243-s001.pdf]

Supplementary Materials: Impact of Dust from Ore Processing Facilities on Rain Water Collection Tanks in a Tropical Environment—The Obvious Source "ASin't Necessarily So"

Brian Gulson, Michael Korsch and Anthony Bradshaw

Analytical Methods

General Methodology

Pb isotope ratios for 29 samples from the Qld EPA were measured by thermal ionization mass spectrometry (TIMS). The dissolution of samples and the separation of Pb from the sample matrices were done in Class 350 clean rooms and Class 3.5 exhaust clean hoods. Only ultra clean reagents were used, with Pb contents less than 8 ppt. Materials coming into contact with the samples were polypropylene or Teflon PFA. After sample dissolution, the Pb was separated on a selective resin column and then evaporated onto rhenium filaments for mass spectrometry. Ratios were measured on a VG Sector 54 Thermal Ionization Mass Spectrometer using software developed by CSIRO.

Because of a phenomenon known as time dependent fractionation, the measured values are corrected to an internationally recognized isotopic standard (SRM981). The precisions quoted on the measured ratios are derived from more than 1000 measurements of this standard and can be regarded as quite conservative.

Sample Preparation

Prior to the separation of Pb on the resin column, each type of sample needed a different treatment. To reduce the possibility of inter-sample contamination the amount of Pb being separated was limited to 300 ng. Procedural blanks are normally less than 20 picograms and are not considered significant.

Rainwater Tank Samples: Between 0.5 mL and 3 mL of tank water (equivalent to ~40 ng of Pb) was evaporated to dryness in a clean hood with a small amount of hydrochloric acid.
Roof and Gutter Swipes: These were supplied as digests. Between 5 and 300 μL was evaporated to dryness in a clean hood.
Remote Dust Samples: 0.1 g of dust was weighed into a beaker and then evaporated to dryness with a 1:1 mix of 8 M nitric acid and 8 M hydrochloric acid.
Concentrate Samples: 10–15 mg of sample was dissolved in aqua regia and evaporated to dryness. Hydrochloric acid was added to the Pb concentrate sample and the supernatant was diluted in hydrochloric acid so that around 300 ng was being processed. The Zn concentrate was similarly diluted.
Blank Samples: 25 mL of the solutions were evaporated to dryness with a small amount of nitric acid after spiking with ^{208}Pb. Normally this would need no further purification, but because of visible residue, each blank underwent the column chemistry. The column blanks are <10 picograms and would not have significantly contributed to the measured blank values.

Table S1. Rainfall at KarumbaAirport, 2008.

	Jan.	Feb.	March.	April	May	June	July	Aug.	Sept.	Oct.	Nov.	Dec.
Rainfall (mm)	118.2	292.2	33.5	0	0	0	0	0	0	5	16	31.2
Number rainy days	10	17	4	0	0	0	0	0	0	1	1	4

Table S2. Container Blanks.

Blank Samples		Cadmium (μg/L)	Lead (μg/L)	Arsenic (μg/L)	Zinc (mg/L)
CIBJB1008-W7	Field Blank Sample	<1	<5	<5	<0.004
PSTB1008-BB1	Container Blank 1 (Sealed Bottle ex SGS Lab in Cairns)	<1	<5	<5	0.029
PSTB1008-BB2	Container Blank 2 (Sealed Bottle ex GC Lab Brisbane)	<1	<5	<5	<0.004

The samples are sample containers containing RO water and the HNO_3 preservative that was used in sampling.

Table S3. Estimated composition of the concentrate products (Source: OZ Minerals).

Component	% Pb	% Zn	% S	% Fe	% As	% Cd
**Zinc Concentrate**						
Average	1.34	58.12	26.63	1.29	0.005	0.137
Maximum	2.33	59.62	30.23	2.34	0.011	0.19
Minimum	0.61	56.13	24.59	0.67	0.004	0.085
**Lead concentrate**						
Average	68.81	4.10	13.03	0.82	0.015	0.013
Maximum	74.61	6.83	14.37	1.57	0.020	0.030
Minimum	62.00	2.87	12.02	0.44	0.008	0.007

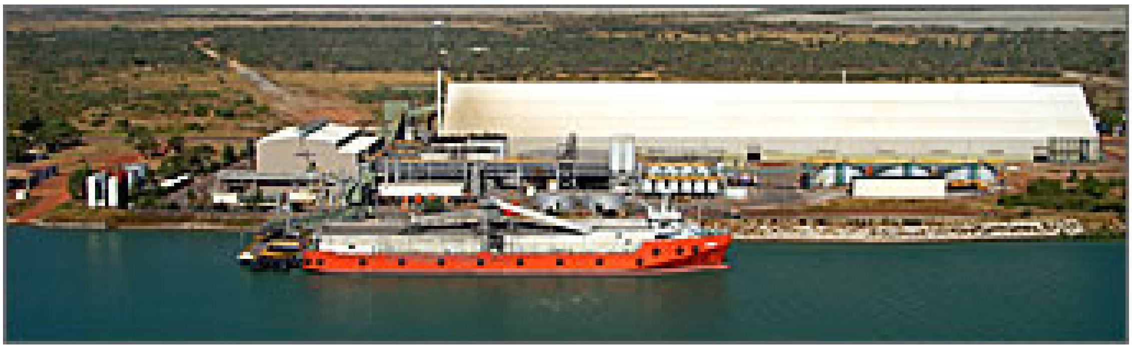

Figure S1. OZ Mineral's Port Site Processing, Storage and loading Facility located beside Norman River at Yappar Street, Karumba.

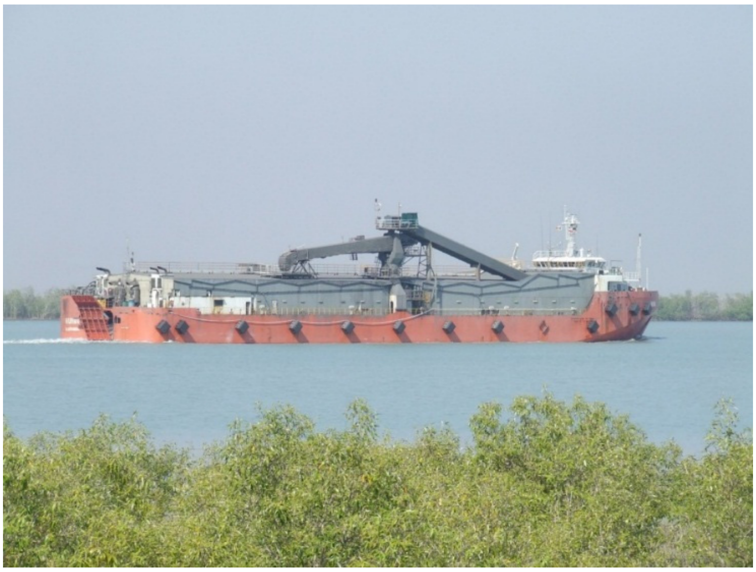

Figure S2. MV Wunma steaming out of Norman River to offshore anchorage where concentrates are transferred to export vessels.

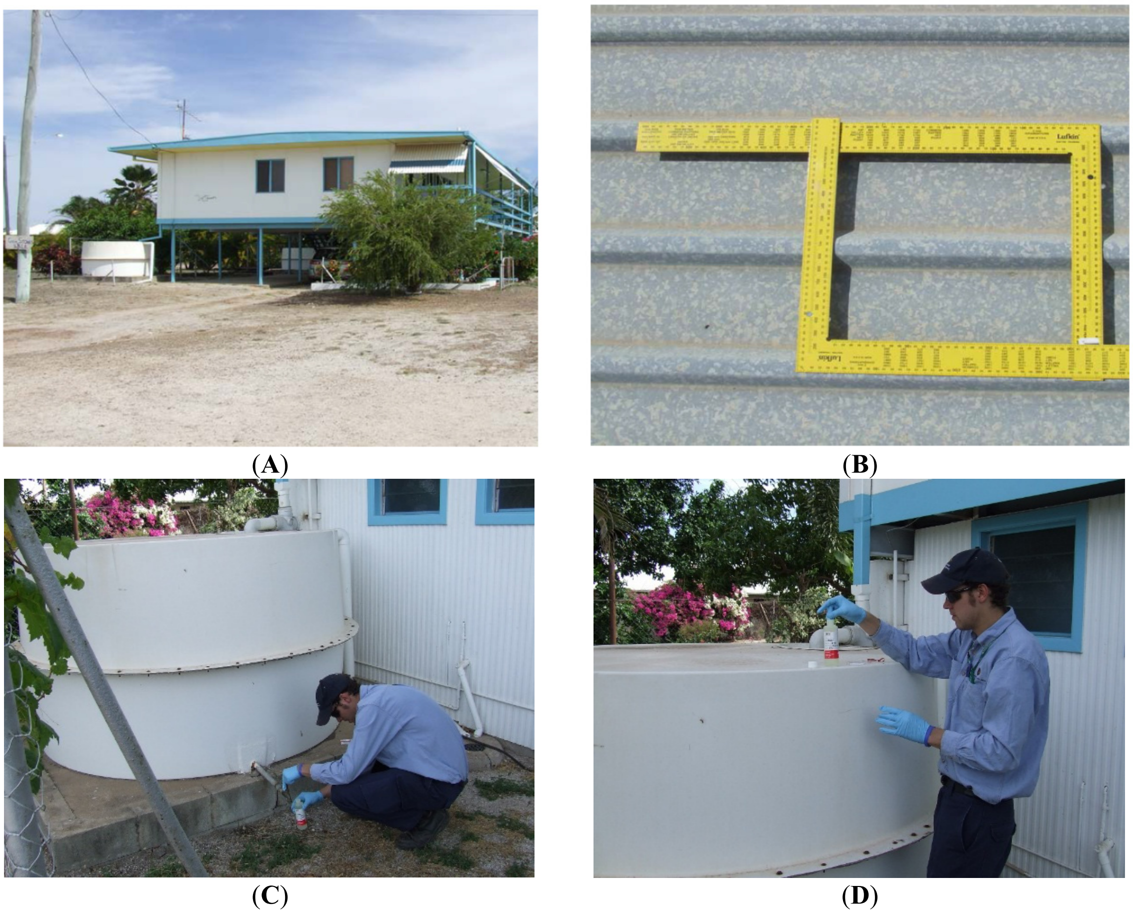

Figure S3. (**A**) 4 Ward Street Karumba point tank and house; (**B**) Roof at 4 Ward Street; (**C**) Tank at 4 ward Street, Fibreglass discharging through galvanised water pipe and brass tap fitting; (**D**) Checking pH of sample following addition of HNO_3 preservative.

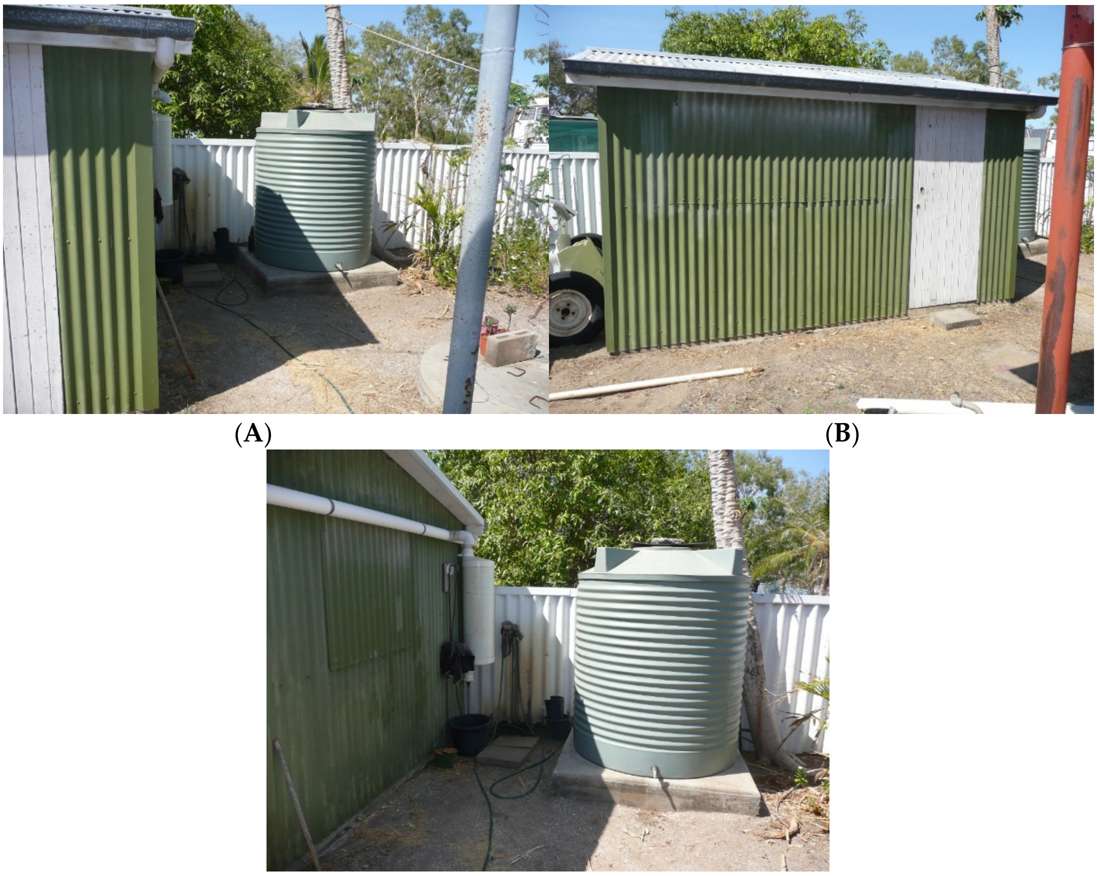

(**A**) (**B**)

(**C**)

Figure S4. 12 Riverview Drive Karumba. (**A**) PVC tank; (**B**) Colorbond roof; (**C**) PVC downpipes and tank.

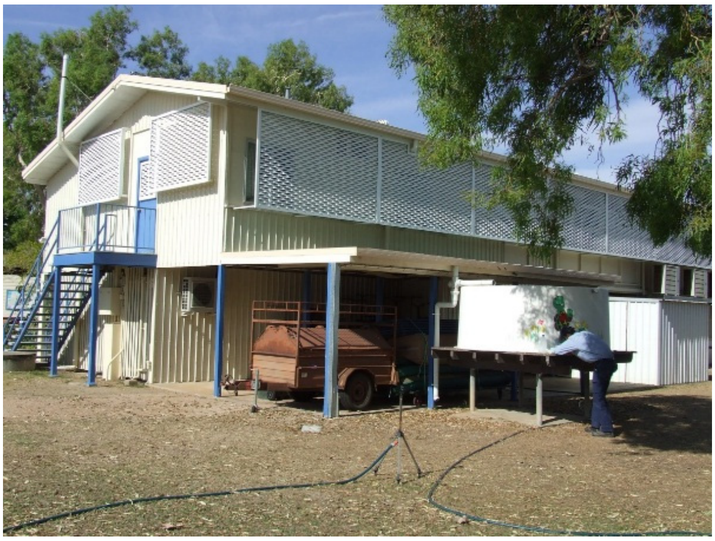

Figure S5. Rain water tank at Karumba School.

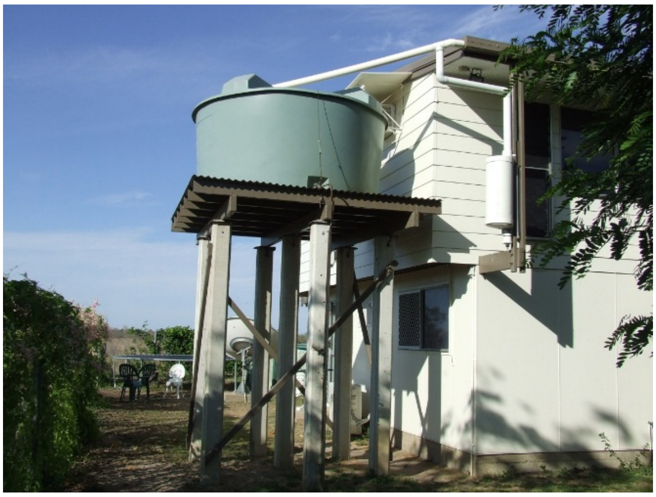

Figure S6. Rainwater tank council depot.

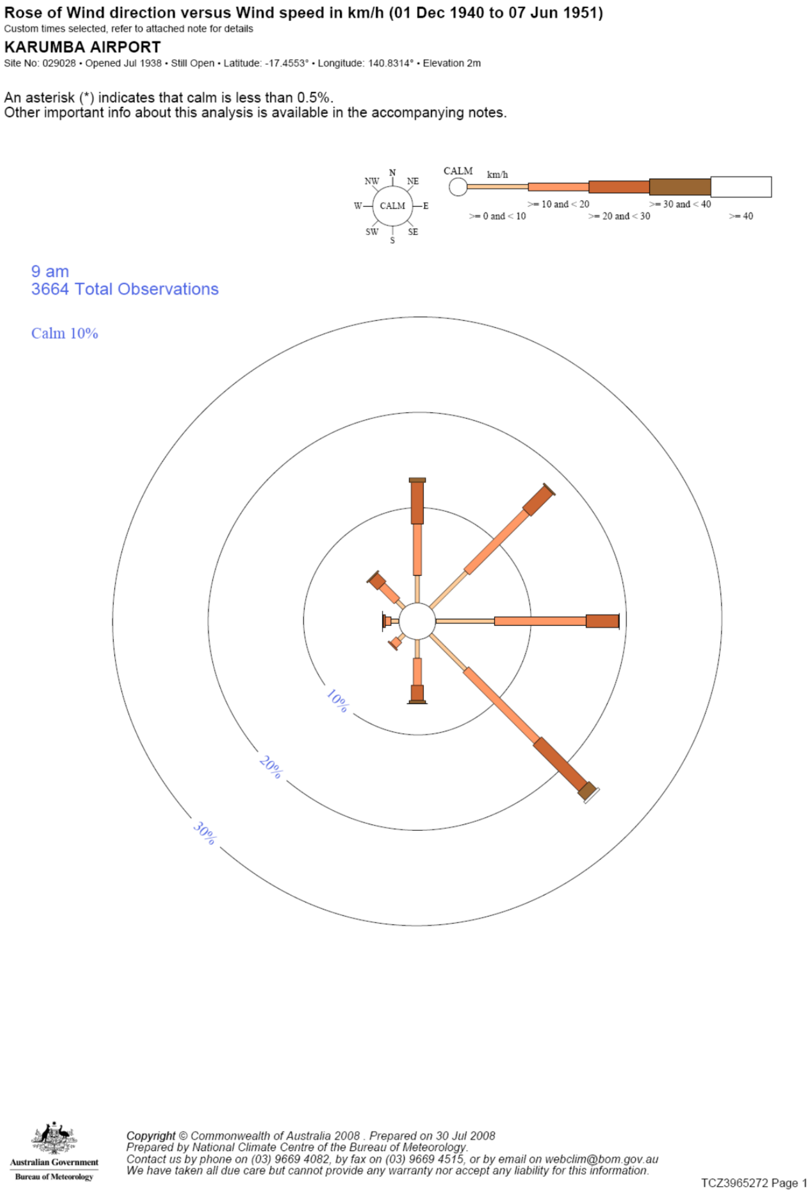

Figure S7. Summary of 9 a.m. Wind Data for Karumba Airport.

Rose of Wind direction versus Wind speed in km/h (01 Dec 1940 to 07 Jun 1951)
Custom times selected, refer to attached note for details

KARUMBA AIRPORT
Site No: 029028 • Opened Jul 1938 • Still Open • Latitude: -17.4553° • Longitude: 140.8314° • Elevation 2m

An asterisk (*) indicates that calm is less than 0.5%.
Other important info about this analysis is available in the accompanying notes.

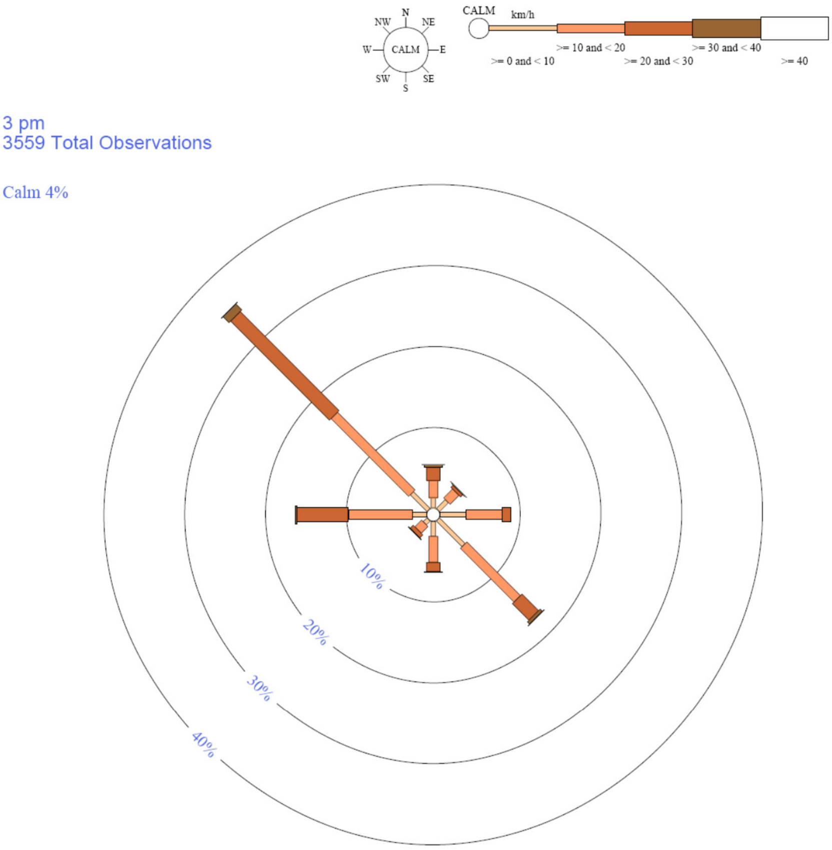

Australian Government
Bureau of Meteorology
Copyright © Commonwealth of Australia 2008 . Prepared on 30 Jul 2008
Prepared by National Climate Centre of the Bureau of Meteorology.
Contact us by phone on (03) 9669 4082, by fax on (03) 9669 4515, or by email on webclim@bom.gov.au
We have taken all due care but cannot provide any warranty nor accept any liability for this information.
TCZ3965272 Page 1

Figure S8. Summary of 3 p.m. Wind Data for Karumba Airport.

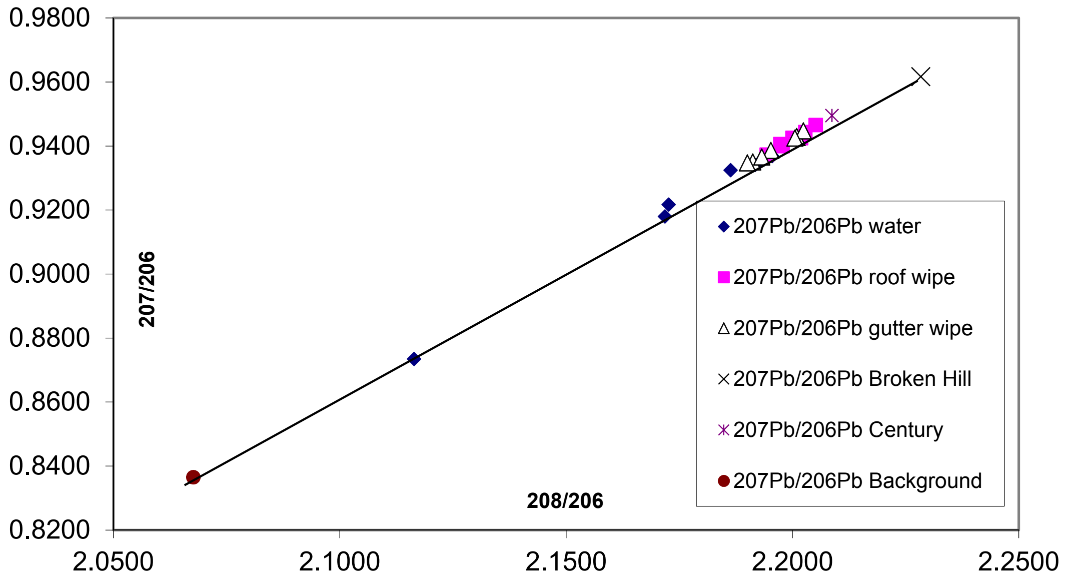

Figure S9. Isotopic ratio plot of the major isotopes $^{207}Pb/^{206}Pb$ *versus* $^{208}Pb/^{206}Pb$ showing the lack of resolution in data using this plot.

© 2016 by the authors; licensee MDPI, Basel, Switzerland. This article is an open access article distributed under the terms and conditions of the Creative Commons by Attribution (CC-BY) license (http://creativecommons.org/licenses/by/4.0/).
